# Family history of the cancer on the survival of the patients with gastrointestinal cancer in northern Iran, using frailty models

**DOI:** 10.1186/1471-230X-11-104

**Published:** 2011-10-01

**Authors:** Mahmoodreza Ghadimi, Mahmood Mahmoodi, Kazem Mohammad, Hojjat Zeraati, Mahboobeh Rasouli, Mahmood Sheikhfathollahi

**Affiliations:** 1 Department of Epidemiology and Biostatistics, School of Public Health, Tehran University of Medical Sciences, Tehran, Iran

**Keywords:** Gastrointestinal tract cancer, Survival analysis, Parametric models, AIC, Frailty models

## Abstract

**Background:**

Gastrointestinal (GI) tract cancer is one of the common causes of the mortality due to cancer in most developing countries such as Iran. The digestive tract is the major organ involved in the cancer. The northern part of the country, surrounded the Caspian Sea coast, is well known and the region with highest regional incidence of the GI tract cancer. In this paper our aim is to study the most common risk factors affecting the survival of the patients suffering from GI tract cancer using parametric models with frailty.

**Methods:**

This research was a prospective study. Information of 484 cases with GI cancer was collected from Babol Cancer Registration Center during 1990-1991. The risk factors we studied are age, sex, family history of cancer, marital status, smoking status, occupation, race, medication status, education, residence (urban, rural), type of cancer, migration status (indigenous, non-native). The studied cases were followed up until 2006 for 15 years. Hazard ratio was used to interpret the death risk. The effect of the factors in the study on the patients survival are studied under a family of parametric models including Weibull, Exponential, Log-normal, and the Log-logistic model. The models are fitted using with and without frailty. The Akaike information criterion (AIC) was considered to compare between competing models.

**Results:**

Out of 484 patients in the study, 321 (66.3%) were males and 163 (33.7%) were females. The average age of the patient at the time of the diagnosis was 59 yr and 55 yr for the males and females respectively. Furthermore, 359 (74.2%) patients suffered from esophageal, 110 (22.7%) patients recognized with gastric, and 15 (3.1%) patients with colon cancer. Survival rates after 1, 3, and 5 years of the diagnosis were 24%, 16%, and 15%, respectively. We found that the family history of the cancer is a significant factor on the death risk under all statistical models in the study. The comparison of AIC using the Cox and parametric models showed that the overall fitting was improved under parametric models (with and without frailty). Among parametric models, we found better performance for the log-logistic model with gamma frailty than the others. Using this model, gender and the family history of the cancer were found as significant predictors.

**Conclusions:**

Results suggested that the early preventative care for patients with family history of the cancer may decrease the risk of the death in the patients with GI cancer. The gender appeared to be an important factor as well so that men experiencing lower risk of death than the women in the study. Since the proportionality assumption of the Cox model was not held (p = 0.0014), the Cox regression model was not an appropriate choice for analysing our data.

## Background

Cancer is known as one of the major causes leading to many disorders, death, and disabilities worldwide [1,2]. Cancer has affected increasingly the human population during the past decades so that considerable amount of health care resources have been allocated to diminish its side effects [3]. It is predicted to become the leading cause of death in many developed and developing countries such as Iran [1,4].

Esophageal, Stomach, and Colorectal cancers are three most common types of cancer among Iranian population [5]. *The *Northern part, located on the Caspian coast has been reported as the main area of the county dominated by *Gastrointestinal (GI) Malignancies *tract cancer [6,7]. Out of 70.4 million of the country's population, almost 50,000 new cases of cancer are reported each year. In more than 38% cases, the GI tract is partially or completely affected.

Stomach, esophageal, and colon cancer are three most common types of cancers reported among males. For females, the breast cancer should be added to this list [8,9]. Cancer is the third most common cause of death in Iran. This accounts for 14% of the total mortality of which the GI cancer accounts for approximately half (44.4%) of all cancers related deaths [10,11]. Unfortunately, the GI cancer in Iran is diagnosed when the disease is in its developed phase and hence the patience the available therapies treatment are less effective to cure the patience [1,11]. Practically, the early diagnosis of the GI tract cancer gives more chance to the patients to recover from the discomfort.

Survival data are often modeled using the Cox proportional hazards model which estimates the covariate effects as the log hazard ratios. This model is free of estimating the baseline hazards for the model. However, since the hazard function is directly related to the time course of the disease, its behavior may be of medical interest. The baseline hazard rate can help us to understand the common history of the disease respect to the hazard rate changing over time [12,13]. Cox's semi-parametric regression model [14] is frequently used to analyse the survival data. Alternatively the fully parametric models such as Weibull, Log-Logistic and Log-Normal models can be used [15,16]. They can offer a gain that may not be obtained under Cox's model. Efron [17] and Oakes [18] showed asymptotically that under certain circumstances, parametric models can lead to more efficient estimates of the parameter.

In survival analysis, to model the data in which the mortality reaches a peak and then starts to decline, a model with a non-monotonic (hump-shaped) failure rate can be used. This is the case with our data we use in this paper. In order to capture efficiently this property of data, the Log-logistic and Log-normal model are often used [13]. However, If the issue of outliers is not major, the Log-logistic model can be used to approximate the Log-normal model. Moreover, for censored data, the Log-logistic model has a simple hazard form and survival function [12,13]. For these reasons, we use the Log-logistic function in this paper for analyzing our data.

The aforementioned pattern for hazard function was the case in our study. Hazard function increased slowly until after a while started to decline. Because of this pattern in our data Cox, Weibull, and Exponential models are not appropriate ones and as was said in above Log logistic model seems better as results of our findings verified the issue.

It is assumed that for the unique covariate inputs, the survival function under the Cox proportional hazards and parametric models is the same for subjects. However, the data may present extra-variation due to the unobserved factors. In this study, we collected data on all possible factors we thought might influence the patient's survival.

A model becoming increasingly popular for modeling the multi-level individual survival times is frailty model. A frailty is an unobserved random effect shared by subjects within a sub-group. Frailty models are also used to capture the overdispersion in univariate survival studies. In this paper, the frailty refers to the effect of the unobserved factors on the subject's survival. Ignoring frailty may lead to the biased survival estimates. The overdispersion is modeled using a latent multiplicative effect on the hazard, or frailty. A gamma or inverse-Gaussian distribution is commonly used to model the frailty [12,19-22]. Thus, the hazard of a population is interpreted as the mean of individual hazards among the survivors. Frail individual with notable values of frailty will tend to die sooner [19]. The frailty (random effect) can be integrated out (in closed form or by numerical or stochastically integration, depending on the frailty distribution) to get a likelihood function not depending on unobserved quantities [19].

By the expectation is conditional on being at risk at time point t, it mention averaging over a subset of the original population. Therefore, relative weights for hazards with high frailty become smaller as time goes by, corresponding to high mortality. An important implication is that studies of human aging based on cohort mortality data may be systematically biased or based on erroneous functional forms [19]. The aim of the this paper is to investigate the factors influencing the survival of the patients with GI tract cancer using parametric models with frailty. We also compare our results with that of achieved under the Cox model.

## Methods

This survey was a prospective study. The total number of 484 patients with developed GI tract cancer registered at the Babol Cancer Registration Center during 1990-1991. They then followed up for 15 years until 2006. The socio-demographic and clinical data obtained using questionnaire and the patients' clinical records. Written informed consent from patients was obtained prior to entering the study. Patients completed a questionnaire that assessed satisfaction with the informed consent procedure. Also to maintain patient privacy, all records were coded with a unique project identifier prior to transmission to the data collection. The study was confirmed by the Ethics Committee of Tehran University of Medical Sciences. The factors we consider in our study are age at diagnosis, gender, place of residence, province, type of cancer, method of cancer detection, family history of cancer, education, job, marital status, cigarette smoking, ethnicity, migration status, drug use.

A multivariate parametric regression model (with and without frailty) was developed to analyse the prognostic factors related to the longevity of patients. To compare the different parametric models and their efficiency the Akaike Information Criterion (AIC) [23], Cox-Snell, and deviance residual plots were used. The AIC was considered to assess the general goodness of fit of the statistical models. The lower value of the AIC, the better model to fit the data. Hazard rate (HR) [12,24] was used to interpret the death risk of the parametric models. For the statistical analysis, the statistical software SAS 9.1 and STATA 8.0 were used. The values less than 0.05 for probability, p ≤ 0.05, was defined as the level of our statistical significance.

## Results

Out of 484 initial patients with developed GI cancer, 321 (66.3%) were men and 163 (33.7%) women. The mean ± standard deviation of age at diagnosis was 58.26 ± 10.9 years and the median survival time was found 9.1 months. The estimated survival rates in 1, 3, and 5 years after diagnosis were 0.24, 0.16, and 0.15 respectively. The type of cancer in these patients was as follows: esophageal (74.2%), stomach (22.7%) and colon (3.1%) (Table 1). During the following up, the total number of 426 (88.0%) deaths were observed (non-censored observations) and 88 (12.0%) patients survived or exact details of their survival status were not available (Loss to follow up)(right censored observations).

**Table 1:** Characteristics of patients with Gastrointestinal tarct cancer diagnosis

Characteristic	n (%)
Gender	
Male	321 (66.3)
Female	163 (33.7)
Place of residence	
Rural	256 (52.9)
Urban	228 (47.1)
Province	
Mazandaran	288 (59.5)
Golestan	196 (40.5)
Type of cancer	
Esophageal	359 (74.2)
Stomach	110 (22.7)
Colorectal	15 (3.1)
Method of cancer detection	
Clinical diagnosis	35 (7.2)
Direct endoscopy and biopsy	39 (8.1)
Conventional chest x-ray	410 (84.7)
Family history of cancer	142 (29.3)
Education	
Literate	52 (10.7)
Illiterate	432 (89.3)
Job	
Farmer	252 (52.1)
Employee	7 (1.4)
Others	225 (46.5)
Marital status	
Married	459 (94.8)
Single	25 (5.2)
Cigarette smoking	215 (44.4)
Ethnicity	
Aryan	327 (67.6)
Gilak	12 (2.5)
Torkaman	100 (20.7)
Others	45 (9.2)
Migration status	
Native	430 (88.8)
Non-native	54 (11.2)
Drug use	119 (24.6)

According to the fact that the proportionality assumption of Cox model was not met in our data (p = 0.0014), using Cox regression was not suitable, even adding frailty term (with gamma and inverse-Gaussian) in to Cox model, proportionality assumption was ever violated and there was no remedy in the violation of the PH assumption. Thus Cox model was omitted from study.

**Figure 1:**
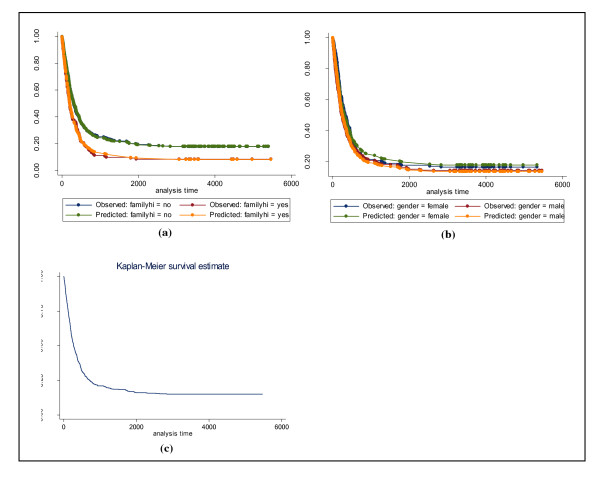
**Survival curve of GI tract cancer patients using Kaplan-Meier method**. (a), (b) Kaplan-Meier estimates of the survival curves for GI tract cancer data separated by family history of cancer and gender, respectively. (c) Kaplan-Meier overall survival curves.

The Kaplan-Meier estimates of the survival functions for the gender and the family history of the cancer are given in the Figure 1.

**Figure 2:**
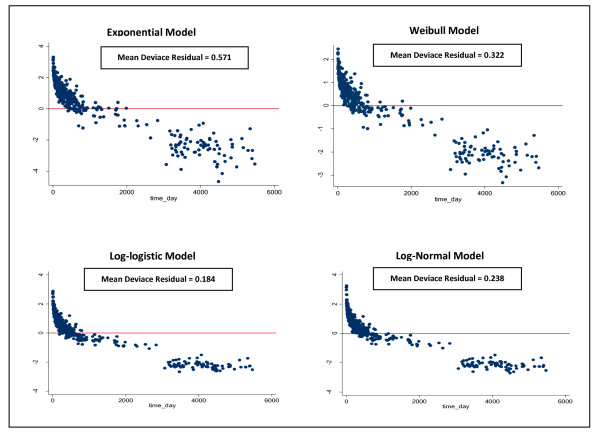
**Deviance residuals to evaluate model fit of parametric models**. In this plot, the deviance residual is large for short survival times and then decreases with time. This pattern suggests that the log normal and log logistic models with gamma frailty are better than other both models (The log logistic model has the lowest mean deviance residual with respect to other models).

**Figure 3:**
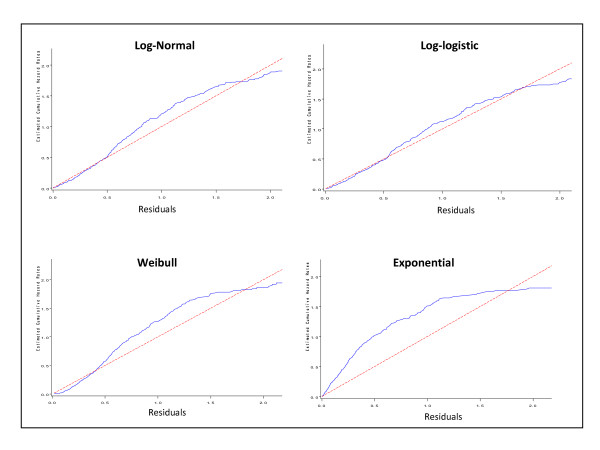
**Cox-Snell residuals obtained from fitting various survival models with gamma frailty to the GI tract cancer data**. The panels indicate the Cox-Snell residuals (together with their cumulative hazard function) obtained from fitting different parametric models to the same data via maximum likelihood estimation.

Figures 2, 3 plots the Cox-Snell and deviance residuals under the parametric models; log-normal, log-logistic, and Weibull model. In overall, the plots show smaller residuals using parametric models and therefore we may conclude they have better performance than the Cox model. Furthermore, the parsimonious of the Cox-Snell residuals under the log-normal and log-logistic model with gamma frailty to the 45 degrees line in Figure 3 confirms these models provide better fitting to our data. It can be also seen that the log-logistic model has better performance over the log-normal model. The weak performance of the Weibull model which assumes the proportional hazards can be due to the violation assumption of the proportional hazards.

The similar conclusion can be obtained by using AIC. The AIC of each model in the study is given in Table 2. The best scores are achieved under the log-logistic model. The Weibull model is the next best model followed by the log-normal. Table 2 also suggests the log-logistic with gamma frailty as the most efficient model for our data.

**Table 2:** Overview of the Akaike Information Criterion Scores

	AIC	Rank
Without Heterogeneity		
		
Exponential	2127.24	4
Weibull	1918.18	3
Log-normal	1800.44	2
Log-logistic	1786.94	1
	**AIC**	**Rank**
**Gamma Heterogeneity**		
		
Exponential	**1780.94**	**4**
Weibull	**1739.86**	**2**
Log-normal	**1746.16**	**3**
Log-logistic	**1717.84**	**1**

	**AIC**	**Rank**
**Inverse Gaussian Heterogeneity**		
		
Exponential	**1851.98**	**4**
Weibull	**1851.58**	**2**
Log-normal	**1759.78**	**3**
Log-logistic	**1725.02**	**1**

Table 3 reports the detailed results of the multivariate analysis for the parametric models with and without frailty based on the HR for each variable. Results of the multivariate analysis show that the family history of the cancer appears a significant factor in all fitted models. This implies that patients with the family history of the cancer are less survived than others.

**Table 3:** Multivariate model of parametric models with and without frailty

**variables**	**Exponential**			**Weibull**			**Log-normal**			**Log-logistic**		
	**HR (P-value)**			**HR (P-value)**			**HR (P-value)**			**HR (P-value)**		
	
	**without frailty**	**Gamma frailty**	**Inv-Gussian frailty**	**without frailty**	**Gamma frailty**	**Inv-Gussian frailty**	**without frailty**	**Gamma frailty**	**Inv-Gussian frailty**	**without frailty**	**Gamma frailty**	**Inv-Gussian frailty**
Age(years)	1.005 (0.296)	1.01 (0.291)	1.01 (0.244)	1.01 (0.316)	1.01 (0.172)	1.01 (0.253)	1.01 (0.252)	1.01 (0.268)	1.01 (0.248)	1.01 (0.269)	1.01 (0.206)	1.01 (0.188)
Gender	1.12 (0.459)	1.32 (0.232)	1.25 (0.324)	1.11 (0.469)	1.47 (0.059)	1.22 (0.348)	1.37 (0.152)	1.64 (0.011)*	1.61 (0.022)*	1.28 (0.245)	1.45 (0.041)*	1.47 (0.042)*
Place of residence	1.15(0.199)	0.99 (0.945)	1.07 (0.668)	1.07 (0.514)	0.95 (0.714)	1.06 (0.697)	1.02 (0.901)	0.96 (0.768)	0.98 (0.891)	0.99 (0.992)	0.92 (0.492)	0.93 (0.568)
Province	0.76(0.069)	0.96 (0.814)	0.77 (0.132)	0.79(0.050)*	1.09 (0.611)	0.79 (0.161)	0.87 (0.412)	1.06 (0.709)	1.01 (0.989)	0.93 (0.669)	1.12 (0.393)	1.12 (0.453)
Type of cancer	0.87 (0.209)	0.95 (0.797)	0.88 (0.420)	0.9 (0.328)	0.99 (0.968)	0.88 (0.432)	0.93 (0.623)	1.05 (0.728)	1.02 (0.921)	0.94 (0.715)	1.03 (0.835)	1.02 (0.901)
Family history of cancer	1.85(< 0.001) *	1.69 (0.004)*	1.92 (< 0.001)*	1.54 (< 0.001)*	1.49 (0.009)*	1.82 (< 0.001)*	1.82 (0.001)*	1.47 (0.013)*	1.56 (0.007)*	1.72 (0.001)*	1.39 (0.016)*	1.43 (0.013)*
Education	0.6 (0.004)*	0.63 (0.114)	0.6 (0.054)	0.69(0.036)*	0.74 (0.213)	0.62 (0.059)	0.62 (0.071)	0.73 (0.201)	0.7 (0.150)	0.6 (0.060)	0.79 (0.307)	0.77 (0.270)
Marital status	1.61 (0.063)	1.64 (0.224)	1.75 (0.139)	1.45 (0.156)	1.49 (0.222)	1.69 (0.150)	1.67 (0.180)	1.35 (0.379)	1.47 (0.272)	1.67 (0.177)	1.31 (0.361)	1.39 (0.305)
Cigarette smoking	1.35 (0.014)*	0.99 (0.989)	1.23 (0.263)	1.2 (0.109)	0.9 (0.533)	1.2 (0.289)	1.06 (0.718)	0.84 (0.306)	0.9 (0.528)	1.03 (0.871)	0.85 (0.298)	0.87 (0.363)
Migration status	1.40 (0.047)*	1.35 (0.266)	1.43 (0.154)	1.23 (0.194)	1.37 (0.194)	1.39 (0.170)	1.45 (0.157)	1.35 (0.191)	1.41 (0.157)	1.35 (0.227)	1.28 (0.267)	1.3 (0.238)
Drug use	0.95 (0.727)	1.11 (0.624)	1.01 (0.958)	0.97 (0.837)	1.07 (0.674)	1.01 (0.944)	1.06 (0.758)	1.11 (0.561)	1.1 (0.614)	1.11 (0.598)	1.09 (0.618)	1.09 (0.637)
Method of cancer detection (Direct endoscopy and biopsy)	0.64 (0.023)*	0.89 (0.751)	0.73 (0.292)	0.76 (0.259)	1.11 (0.814)	0.76 (0.319)	0.84 (0.573)	1.11 (0.712)	1.06 (0.839)	0.85 (0.613)	1.07 (0.790)	1.08 (0.775)
Method of cancer detection (Clinical diagnosis)	0.39 (< 0.001)*	0.57 (0.142)	0.44 (0.011)*	0.51 (0.003)*	0.70 (0.495)	0.46 (0.013)*	0.56 (0.071)	0.95 (0.878)	0.84 (0.586)	0.52 (0.059)	0.93 (0.795)	0.88 (0.688)
Job (Employee)	1.06 (0.895)	0.85 (0.829)	0.95 (0.937)	0.98 (0.972)	0.69 (0.758)	0.93 (0.917)	1.13 (0.856)	1.89 (0.445)	1.71 (0.494)	0.85 (0.814)	0.84 (0.773)	0.83 (0.772)
Job (Others)	1.01 (0.926)	0.98 (0.918)	1.01 (0.944)	0.99 (0.996)	0.99 (1.00)	1.01 (0.972)	1.04 (0.832)	1.08 (0.638)	1.09 (0.618)	0.97 (0.873)	0.99 (0.936)	0.99 (0.967)
Ethnicity (Gilak)	1.35 (0.329)	0.53 (0.211)	0.87 (0.783)	1.08 (0.806)	0.22 (0.041)*	0.85 (0.745)	0.62 (0.351)	0.39 (0.027)*	0.42 (0.061)	0.58 (0.224)	0.43 (0.020)*	0.42 (0.023)*
Ethnicity (Torkaman)	1.33 (0.089)	1.30 (0.327)	1.35 (0.255)	1.23 (0.202)	1.34 (0.434)	1.32 (0.255)	1.24 (0.414)	1.04 (0.878)	1.04 (0.881)	1.34 (0.231)	1.23 (0.293)	1.21 (0.351)
Ethnicity (Others)	1.18 (0.377)	1.39 (0.267)	1.21 (0.490)	1.12 (0.517)	1.79 (0.160)	1.21 (0.476)	1.23 (0.469)	1.26 (0.344)	1.21 (0.465)	1.39 (0.229)	1.52 (0.062)	1.48 (0.092)

* Significant at 0.05 level

HR, Hazard rate

Gender is significant under the log-normal and log-logistic with gamma frailty model but not significant factor under other models. This indicates that the level of the death risk due to GI cancer was reduced significantly for the women in the study during the following up period.

None of the parametric models suggests age, residence, province, type of cancer, methods of cancer diagnosis, educational level, occupation, smoking, ethnicity, migration status and drug use as a significant prognostic factors.

## Discussion

GI tract cancer is one of the most common types of cancer in Iran [10]. The cancer is a particularly devastating form of cancer with a relatively low survival rate, and people generally will not live a long time after diagnosis. Several factors known in various studies as influencing prognosis factors and have been introduced [25-33].

In the literature, there are many studies on the field of cancer, but researchers tend to examine the effects of covariates on patients survival using Cox regression model instead of parametric ones. A systematic study on Cancer Journals shows that only in 5% of studies of cancer in which Cox regression model is used the assumptions of the model have been investigated [34]. If presumptions are not met, results of Cox model are seriously under question. As an alternative, parametric models such as log-normal, log logistic, Weibull, and exponential can be employed. The only assumption of parametric models is that the variable time follows a specific distribution [13,24].

In this paper we aimed to study the possible relationship between the survival of the patients with GI tract cancer and several most common prognosis factors such as age at diagnosis, gender, place of residence, province, type of cancer, method of cancer detection, family history of cancer, education, job, marital status, cigarette smoking, ethnicity, migration status, drug use.

We found gender and the family history of the cancer significant prognostic factor. This supports the past studies reporting better survival for women with developed GI tract cancer [1,35-37] and the family history of the disease as a significant factor [38,39].

Statistical assessment using AIC of the studied models showed that the log-logistic model with gamma frailty will describe our data better.

Due to their better performance, our intension is to use parametric models. However, the efficiency of the parametric models is greatly affected by the volume of the censored observations. For having sensible results, it is recommended that the percentage of the censored data should not be more than 50%[40]. This condition is satisfied with our data as they consist of 15% censored observations.

Nardi et al. [40] compared the performance of the Cox model and some parametric models. They used normal-deviate residuals [41] to evaluate the assumptions of parametric models. They also studied Weibull model based on the estimated variation of the parameter rate criteria, and concluded that the Weibull was the superior model. In our study, we found the log-logistic model to have better performance than the other models in the study.

By a simulation study, HRbe et al. [42] compared the Cox regression model and the accelerated failure time (AFT) models. They used the proposed method by Stute [43] for fitting linear regression models with right-censored data. Their results showed that whatever the proportional hazards assumption is violated or not, the log logistic, log-normal, and the Stute models are more efficient than the Cox model.

Bradburn et al. [44] evaluated the adequacy of some parametric models and the Cox proportional hazards model using model's residuals and the AIC. They found that the generalized gamma model and parametric models achieved both a higher log-likelihood and a lower AIC.

For the Cox and parametric models, the hazard function may depend on the unknown or latent factors which can lead to the biased estimates of the regression coefficients [19,45]. To overcome this issue we used the frailty models. In fact these models are used to explain the random variation of the survival function that may exist due to unknown risk factors such as genetic factors and other environmental factors [19,22,45-47].

Random effects models are known as the frailty models in the survival analysis. These models, widely studied in the 1990's, are relatively new in the survival field and are currently mainly under investigations, but technical problems in estimating the parameters of frailty models made to be used less compared to the Cox model. Using frailty to model the extra-variation in univariate lifetime data goes back to the work of Vaupel et al. [48]. Henderson and Oman [49] in a theoretical method revealed that in case of non-use of frailty model when there is frailty effect bias may occur in the estimates of regression coefficients. Schumacher et al. [50] showed that ignoring an important factor can lead to lower estimations of the relative risk by the fitted models. Keiding et al. [51] showed how removing one of the two explanatory variables might increase the variance of the hazard function and biased estimation of other coefficients in the fitted model. They suggested using AFT models to handle the effect of unobserved variables.

According to our findings, log logistic model with gamma frailty is more suitable statistical model in survival analysis in patients with GI cancers rather than other parametric models.

## Conclusions

Our study showed that the gender and the family history of the cancer were two factors that can significantly affect the lifetime of the patients with GI tract cancer.

According to our findings the early recognition of family history of cancer and, in consequence, awareness of family members to consider the possibility of family screening may result in a decrease in death rate due to GI tract cancer.

Furthermore, we found that the death risk of the GI tract cancer for the men was significantly lower than the women. We also recommended to use the log-logistic with gamma frailty model, to evaluate the effects of the prognostic factors on the developing the GI tract cancer.

## Limitation

One of the limitations of this study was the lack of an efficient recording medical system in the Babool Cancer Registeration Center. Currently there is no any information available for some clinical factors such as the type of esophageal cancer (adenocarcinoma, squamous) and the stage of the disease.

## Competing interests

The authors declare that they have no competing interests.

## Authors' contributions

MG, MM and KM participated in the design of the study and performed the statistical analysis. MS and MR participated in data collection and helped to draft the manuscript. All authors read and approved the final Manuscript.

## Pre-publication history

The pre-publication history for this paper can be accessed here:

http://www.biomedcentral.com/1471-230X/11/104/prepub
